# Identifying alternatives to old age psychiatry inpatient admission: an application of the balance of care approach to health and social care planning

**DOI:** 10.1186/s12913-015-0913-1

**Published:** 2015-07-17

**Authors:** Sue Tucker, Christian Brand, Mark Wilberforce, Michele Abendstern, David Challis

**Affiliations:** Personal Social Services Research Unit, University of Manchester, Crawford House, Booth Street East, Manchester, M13 9QS UK

**Keywords:** UK, Health service planning, Resource allocation, Commissioning, Older people, Mental health, Balance of care, Hospital admission, Community care

## Abstract

**Background:**

Mental health problems in older people are common and costly, posing multiple challenges for commissioners. Against this backdrop, a series of initiatives have sought to shift resources from institutional to community care in the belief that this will save money and concurs with user preferences. However, most of this work has focused on the use of care home beds and general hospital admissions, and relatively little attention has been given to reducing the use of mental health inpatient beds, despite their very high cost.

**Methods:**

The study employed a ‘Balance of Care approach’ in three areas of North-West England. This long-standing strategic planning framework identifies people whose needs can be met in more than one setting, and compares the costs and consequences of the possible alternatives in a simulation modelling exercise. Information was collected about a six-month cohort of admissions in 2010/11 (*n* = 216). The sample was divided into groups of people with similar needs for care, and vignettes were formulated to represent the most prevalent groups. A range of key staff judged the appropriateness of these admissions and suggested alternative care for those considered least appropriate for hospital. A public sector costing approach was used to compare the estimated costs of the recommended care with that people currently receive.

**Results:**

The findings suggest that more than a sixth of old age psychiatry inpatient admissions could be more appropriately supported in other settings if enhanced community services were available. Such restructuring could involve the provision of intensive support from Care Home Outreach and Community Mental Health Teams, rather than the development of crisis intervention and home treatment teams as currently advocated. Estimated savings were considerable, suggesting local agencies might release up to £1,300,000 per annum. No obvious trade-off between health and social care costs was predicted.

**Conclusions:**

There is considerable potential to change the mix of institutional and community services provided for older people with mental health problems. The conclusions would be strengthened by further studies and the incorporation of evidence about relative outcomes. However, the utility of the approach in challenging established patterns of resource allocation and building local ownership for change is apparent.

**Electronic supplementary material:**

The online version of this article (doi:10.1186/s12913-015-0913-1) contains supplementary material, which is available to authorized users.

## Background

The provision of care for older people with mental health problems poses multiple challenges for commissioners [1, 2]. Although there is recent evidence of a decline in the incidence and prevalence of dementia in developed countries [3, 4], the absolute number of people with dementia in Western Europe is anticipated to double by 2050 [5]. In addition, approximately 15 % of older adults have depression [6], and still others are affected by anxiety, schizophrenia, paranoid states and substance misuse [1].

Mental health problems in old age are thus very common. They also carry high costs, both personal and economic. Many disorders are subject to relapse or of long duration, and every aspect of a person’s functioning can be affected, leading to increased resource use [1, 7]. Indeed, dementia alone is estimated to cost the UK economy £23 billion per year [8]. There is then a considerable incentive to find the most appropriate, effective and efficient ways of caring for this group.

Against this background, recent years have seen the development of a series of initiatives designed to shift resources from institutional to community care, predicated on grounds of cost-effectiveness and user preferences. However, most of these plans have focused on the use of care home and general hospital beds, and surprisingly little attention has been paid to the demand for specialist mental health inpatient services, despite their very high cost [9–11].

### Commissioning services for older people with mental health problems

Following recent reform of the National Health Service (NHS), a network of over 200 Clinical Commissioning Groups are responsible for commissioning secondary and community health care for their local populations in England. Composed in part of General Practitioners, these groups control approximately two-thirds of the NHS budget. Key activities include identifying local needs, setting priorities and exercising budgetary control, encompassing fundamental decisions about the distribution of resources across different service areas, interventions and patient groups [12, 13].

Whilst the complexity of such tasks may appear self-evident, commissioning services for older people with mental health problems presents a number of special problems. These include the broad range of care delivered to this client group and the large number of (health, social, independent and voluntary sector) providers [14]. Despite increasing investment in economic evaluations, there is also a lack of evidence about the relative cost-effectiveness of institutional and non-institutional provision [1, 15], as most evaluations relate to specific treatments or technologies, and are of limited value to planners interested in the costs and outcomes of changes across the wider spectrum of care [16].

Such gaps highlight how important it is for commissioners to access the experience-based knowledge held by people using and providing services [17], and since the early 1990s government policy has placed increasing emphasis on engaging users in local service development [15, 18, 19]. The benefit of involving providers in the strategic planning process has perhaps had a lower profile, although this is a *World Class Commissioning* competency [20]. Indeed, particularly where commissioners might wish to redeploy resources, provider involvement would seem imperative, for past experience has shown that frontline clinicians will only own such changes if they have been fully involved in them. Securing their engagement is, thus, key to success [21, 22].

### The Balance of Care approach

By way of contrast, the Balance of Care (BoC) approach offers planners, providers and commissioners a systematic framework for examining service efficiency by exploring the likely costs and outcomes of changes in the provision of community and institutional services across health and social care as part of the strategic planning process. Originally conceived of as a national policy analysis tool, the general premise is that although resources are scarce, significant amounts of money can be moved from one patient group/service to another so as to increase benefits and/or reduce costs [23, 24].

At the core of this approach is the identification of those patient groups whose needs can be met in more than one location. Hence, although it is generally accepted that for some individuals a particular care setting (e.g., a hospital bed) is the only appropriate location, the approach focuses on identifying those individuals who could be supported in more than one setting (e.g., an inpatient ward or their own home), people described as ‘on the margins of care’. It then assesses the costs and consequences of the alternative options in a simulation modelling exercise, providing an empirically-based framework for challenging the existing distribution of resources. The defining characteristics of BoC studies are, thus, the identification and measurement of those patient characteristics that affect decisions about where best to support them; some means of matching people to the most appropriate care location; a specification of the resources/inputs required; and an examination of the relative costs (and ideally outcomes) of care in different settings [25, 26].

This pragmatic approach has many advantages for commissioners, enabling a mixture of locally relevant data, research findings and experienced practitioners’ opinions to be built into the decision-making process. It accepts that health and social care planning is not wholly evidence-based, but provides a structured framework by which stakeholders can explore the consequences of alternative actions. It also seeks to engage providers in the planning process (building ownership), and, in focusing on patients’ needs, encourages participants to look beyond existing service patterns [27]. Nevertheless, a recent systematic literature review identified just two studies that had applied the BoC framework specifically to the care of older people with mental health problems [28], one of which dated from the early 1990s [29], whilst the other had a relatively small sample [30]. (Further details of the underlying principles of the BoC approach and step-by-step accounts of the process are available elsewhere [25, 26, 28].)

### The development of specialist old age psychiatry services in England

Although the vast majority of older people with mental health problems have always been cared for in their own homes, the first specialist psychogeriatric services in England were generally hospital-based, with beds in long-stay wards and a high proportion of chronically ill patients [31]. Right from the start, however, the discipline took a community orientation reflecting the widespread closure of the asylums [32] and similar shifts in the social care sector, where a series of reforms sought to reduce the use of institutional care and expand the provision of community support including dementia-specific home and day care services and extra care housing [19, 33–35].

By the start of the 21st century localities aimed to offer comprehensive, accessible, responsive, individualised, multidisciplinary, accountable and systematic mental health care via integrated health and social care services [19, 36], with multidisciplinary Community Mental Health Teams (CMHTs) given a central role in providing support for people with severe or complex mental health problems in the community [37]. This was not to suggest that inpatient beds were no longer necessary, however. On the contrary, it is generally agreed that there will always be a significant minority of people who need hospital admission, with the intensive levels of assessment, monitoring and treatment this offers [38, 39]. That said, there *is* considerable concern about the extent of variation in investment and practice [39, 40], for although inpatient admission can offer a safe haven, it can also be a traumatic experience, exacerbating disorientation and behavioural disturbance, and upsetting usual routines [41]. It also accounts for a large proportion of specialist mental health expenditure [42].

### Aims

Against this backdrop, we undertook a BoC study to investigate the mix of services needed by older people with mental health problems in North-West England. Whilst the wider study investigated the potential for substitution between a broad range of institutional and community services, this paper focuses on the use of mental health inpatient beds, and serves as an illustration of the way in which the BoC approach can engage providers in the strategic planning process. It addresses the following key questions:

- Who is admitted to an acute old age psychiatry ward and why?
- Would it be possible to provide more appropriate care for some of these patients, and, if so, who needs what?
- What would this cost, and how would the cost burden change?

Although conducted in just one country, these are questions of international concern, and the planning and service delivery issues they raise are expected to have a resonance for a wider audience including commissioners, providers and policy makers across the developed world.

## Methods

Building on the findings of the aforementioned systematic literature review of the past use of the BoC approach [28], the study applied a refined version of this tool developed by the Personal Social Services Unit over more than 15 years [30, 43, 44]. This is characterized by its firm grounding in the experience and knowledge of front line practitioners (giving validity to complex judgements about the balance of needs and resources), and utilises quantitative and qualitative methods in a sequential mixed methods design. Each of the six main elements is described below.

### 1. Service user profiling

The study was conducted in three geographical areas served by two neighbouring Trusts. Together these had population of approximately 130,000 people aged 65 plus, and a sociodemographic profile similar to England as a whole.

Data on the sociodemographic, functional, clinical and service receipt characteristics of a six month cohort of older people (65+) admitted to acute mental health inpatient care were collected by nominated nursing staff shortly after admission in 2010/11. These included a number of standardised measures [45–49]. A limited amount of information was also collected at discharge, including length of hospital stay. Admissions for planned respite were excluded.

### 2. Case type development

The sample was divided into subgroups of people with similar needs for care on the basis of four variables deemed likely to be important in determining the locus and/or costs of their care [28]. These were: a broad grouping of primary diagnosis; a hierarchy of risk/concern capturing the main reason for admission; a classification of the extent to which individuals displayed behaviours difficult for carers to manage based on a simple additive index; and the presence or absence of a resident carer (Table 1). In combination, these generated 72 different possible sub-groups or ‘case types’. The homeogeneity of the needs of the people represented by each case type was then checked, and in one instance the group was further divided according to individuals’ usual place of residence, home or care home.

**Table 1:** Criteria used in the classification of case types

Dimension	Categories
Primary diagnosis	Organic mental health problem (most commonly dementia)
	Depression or anxiety
	Other mental health problem e.g., schizophrenia
Main reason for admission	Risk of deliberate self-harm
	Other risks i.e., self-neglect, accidental self-harm, abuse/exploitation or falls, or carer stress
	Behaviour management related i.e., need for behaviour management, risk of harming others or care breakdown
	Need for diagnostic assessment, medication review or treatment
Challenging behaviour	Low (behaviour score 0 or 1)
	Medium (behaviour score 2–7, typically including agitation, wandering and/or disturbed sleep)
	High (behaviour score 8–14, typically including resistance to care and/or aggression)
Living situation	Resident primary carer (including care home residents)
	No resident carer

### 3. Vignette formulation

A series of vignettes were formulated to exemplify the most prevalent case types. These were based on real individuals and took the form of brief case histories which systematically incorporated information about the four key variables listed above as well as patients’ mental health history, ability to undertake activities of daily living (ADLs), physical health, cognition, affect and support network. An example of a vignette is provided in an additional file (see Additional file 1).

### 4. Alternative care planning

Local staff from across the care continuum were invited to three care-planning workshops (one per locality) at which the most appropriate ways of meeting the needs of the people depicted in the vignettes were explored. Workshop participants were divided into small multidisciplinary groups, each of which was allocated a subset of vignettes. Working individually, participants first indicated whether it was ‘completely’ (two points), ‘possibly’ (one point) or ‘not’ (no points) appropriate to admit each of the depicted patients to a mental health bed. The points were then totaled and the sums expressed as a percentage of the maximum possible, enabling the case types to be ranked in order of appropriateness for admission.

Working in small groups, participants then specified the care services needed to divert those case types considered least appropriate for inpatient admission from hospital care (the ‘marginal case types’). For the purposes of this exercise, participants were asked to put aside short-term constraints in services and to be creative, whilst remembering that all provision inevitably has funding implications.

### 5. Validation of local decision-making

The case type ratings from the three workshops were combined, and eight acknowledged national experts in the care of older people with mental health problems were asked to review the alternative care plans for the marginal case types and to identify their preferred option. Experts were predominantly old age psychiatrists with extensive clinical and academic experience.

### 6. Cost comparisons

The estimated costs of the care plans favoured by the experts were compared with the costs of the packages of care individuals actually received immediately prior to inpatient admission. A public sector costing approach was employed, focusing on the most important (expensive or commonly incurred) costs borne by mental health and social services. Wherever possible, costs were calculated from data provided by participating agencies. If local unit costs were unavailable, figures were based on national sources [10]. The aggregate annual savings that might be achieved by substituting the preferred community arrangements for inpatient admission were then estimated, drawing on information about the yearly number of admissions in each case type and the likely length of inpatient stay. Further details of the costing exercise are provided elsewhere [50].

### Ethics

Ethical approval for the study was granted by the then Cambridgeshire 3 Research Ethics Committee (reference number 10/H0306/51) and research governance procedures in each participating organisation were fulfilled. All of the information supplied to the research team for this strand of the study was fully anonymized and no contact was made with service users. As such this activity was adjudged to be a mixture of audit and service development for which service user consent was not required.

## Results

### Service user characteristics

Information was collected about 216 admissions, of which the vast majority (96 %) related to people with a single admission episode in the data collection period. Of these, approximately four-fifths lived at home (about half alone and half with others), whilst the remainder lived in care homes. Table 2 details their sociodemographic, functional and clinical characteristics.

**Table 2:** Inpatient admissions: Sociodemographic, functional and clinical profiles by usual residence

Dimension	Categories	Admissions from home		Admissions from care homes		p-value of *χ* ^2^ test
		%	(n)	%	(n)	
Gender	Female	61.2	(109)	54.1	(20)	0.417
	Male	38.8	(69)	45.9	(17)	
Age	65-74	46.6	(82)	22.2	(8)	0.023
	75-84	38.6	(68)	52.8	(19)	
	85+	14.8	(26)	25.0	(9)	
Activities of daily living^a^	Independent	69.3	(124)	24.3	(9)	<0.001
	Minimal help needed	21.8	(39)	43.2	(16)	
	Partially dependent	5.0	(9)	16.2	(6)	
	Very/totally dependent	3.9	(7)	16.2	(6)	
General health status	Very good/excellent	12.6	(22)	2.9	(1)	0.043
	Good	29.1	(51)	31.4	(11)	
	Fair	33.7	(59)	54.3	(19)	
	Poor	24.6	(43)	11.4	(4)	
Primary diagnosis	Organic mental health problem	31.0	(56)	70.3	(26)	<0.001
	Depression or anxiety	50.9	(89)	21.6	(8)	
	Other mental health problem e.g., schizophrenia	17.2	(30)	8.11	(3)	
Indicators of low mood or anxiety	Yes	76.2	(125)	68.6	(24)	0.344
	No	23.8	(39)	31.4	(11)	
Cognition^a^	Intact/only mild impairment	74.0	(125)	41.7	(15)	<0.001
	Moderate impairment	12.4	(21)	19.4	(7)	
	Severe impairment	13.6	(23)	38.9	(14)	
Challenging behaviour^a^	Low	14.0	(25)	2.7	(1)	0.044
	Medium	63.7	(114)	59.5	(22)	
	High	22.3	(40)	37.8	(14)	
Risks^b^	At least one high risk	77.1	(138)	81.1	(30)	0.595
	No high risks	22.9	(41)	18.9	(7)	

^a^Includes small proportions of imputed cases (model-based)

^b^Includes the risk of deliberate self-harm, harming others or self-neglect

Overall, female admissions outnumbered male by three to two. More than four-fifths were under 85, and a similar proportion were nearly or completely independent in ADLs. Three-quarters were in at least fair physical health. People with a primary organic diagnosis (most commonly dementia) made up approximately two-fifths of admissions, whilst approaching half had depression or anxiety. The remainder had another functional mental health problem, such as schizophrenia. The vast majority displayed at least medium levels of challenging behaviour, and over three-quarters had at least one high level risk, highlighting the severity of their needs. Indeed, almost half were described as often agitated/restless; a similar proportion displayed at least occasional delusions/hallucinations/paranoid ideas; and still more were sometimes or often disturbed at night.

Perhaps not surprisingly, admissions from care homes were significantly older (*p* = 0.023) and more dependent (p < 0.001) than admissions from home. They were also more likely to have an organic diagnosis (p < 0.001), to be in worse physical health (*p* = 0.043) and to display high levels of challenging behaviour (*p* = 0.044). Further analysis of the admissions from home (own home) revealed clear differences in the profiles of people with organic and functional diagnoses (not shown). The latter were significantly more likely to be women (71 % vs. 44 %; *p* = 0.001), to be independent (76 % vs. 54 %; *p* = 0.023) and to have indicators of low mood (82 % vs. 65 %; *p* = 0.017), whereas admissions with organic diagnoses displayed more challenging behaviour (40 % vs. 14 % rated as high; p < 0.001). Few differences were seen between admissions with organic diagnoses from home or care homes, albeit the latter were typically older (36 % vs. 18 % in the 85+ age bracket; *p* = 0.039) and more dependent (20 % vs. 7 % rated as very/totally dependent; *p* = 0.031).

### Reasons for inpatient admission

An average (median) of three factors contributed to each admission (maximum eight). The most commonly cited reason for admission was the need for medication review, whilst other factors noted in at least a third of cases were the risk of self-neglect and the need for general diagnostic assessment, behavioural management or assessment of future care (Table 3). However, these were not necessarily the *main* reasons for admission. The most important driver for people with organic diagnoses was the need for behavioural management, closely followed by the risk of harm to others. For people with functional illnesses, the risk of self-harm and the risk of self-neglect were the top two drivers.

**Table 3:** Inpatient admissions: Reasons for admission by broad diagnostic group

Reason	Organic/mixed	Functional	All	p-value of *χ* ^2^ test
	% (*n* = 83)	% (*n* = 128)	%	
At unacceptable risk of deliberate self-harm	10	39	27	<0.001
At unacceptable risk of accidental self-harm	16	11	13	0.316
At unacceptable risk of self-neglect	27	47	39	0.003
At unacceptable risk of falls	10	5	7	0.250
At unacceptable risk of harming others	36	10	20	<0.001
At unacceptable risk of abuse/exploitation	5^a^	7	6	0.514
For general diagnostic assessment	42	38	40	0.573
For behaviour management	54	38	45	0.023
For review of medication	70	61	64	0.185
For other treatment	6	9	8	0.491
For assessment of future care needs/placement	53	23	35	<0.001
To relieve carer stress	34	28	30	0.387
Other breakdown of care	12	6	9	0.141

^a^Cell size n < 5

### Prior service receipt

More than six-tenths of admissions who lived at home received regular informal care, most commonly from their spouse (Table 4). The extent of this varied considerably, however, whilst less than a third had a formal social care package. As might be expected, people with dementia were significantly more likely than people with functional diagnoses to receive both informal (41 % vs. 20 % receiving 21+ hours; *p* = 0.030) and formal (15 % vs. 3 % receiving an intensive care package; *p* = 0.017) social care. By way of contrast, over seven-tenths of the sample had received at least some community mental health input prior to hospital admission, and approximately a sixth had had a previous recent inpatient stay.

**Table 4:** Inpatient admissions from home: Formal and informal support prior to admission

		Organic/mixed diagnosis		Functional diagnosis		p-value of *χ* ^2^ test
Dimension	Categories	%	(n)	%	(n)	
Informal care	None	29.6	(16)	46.9	(52)	0.030
	1-7 hours per week	13.0	(7)	17.1	(19)	
	8-20 hours per week	16.7	(9)	16.2	(18)	
	21+ hours per week	40.7	(22)	19.8	(22)	
Main informal carer^a^	Spouse	65	(26)	49.2	(30)	0.244
	Child (including children-in-law)	30	(12)	39.3	(24)	
	Other	5	(2)	11.5	(7)	
Lives with main informal carer^a^	Yes	74.4	(29)	59.3	(35)	0.126
	No	25.6	(10)	40.7	(24)	
Nature of informal support^a^	Personal and/or physical	60.7	(17)	30.4	(17)	0.008
	Other practical help	39.3	(11)	69.6	(39)	
Formal social support^b^	None or very minor	53.9	(21)	76.8	(76)	0.017
	Intermittent	12.8	(5)	10.1	(10)	
	Limited care package	18.0	(7)	10.1	(10)	
	Intensive care package	15.4	(6)	3.0	(3)	
CMHT input	Yes	79.0	(45)	68.7	(79)	0.158
	No	21.1	(12)	31.3	(36)	
Recent mental health admission	Yes	20.4	(11)	18.2	(20)	0.737
	No	79.6	(43)	81.8	(90)	

^a^Based on applicable cases (i.e., those that received at least one hour of informal care)

^b^Intermittent input less than daily, mainly outside the home; Limited care package 1–9 hours personal care/domestic help/sitting service combined or seven or more meals per week; Intensive care package ≥ 10 hours personal care/domestic help/sitting service combined per week

### Case type distribution

Of the 73 case types used to characterise the sample, 47 were populated. Together these represented 98 % of the sample. As expected, some combinations of characteristics were more prevalent than others, and only those case types representing four or more admissions at the point of commencing analysis (halfway through the data collection process) were depicted in vignettes (Table 5).

**Table 5:** Characteristics of the 17 inpatient case types depicted in the vignettes

Case type	Broad diagnosis	Main reason for admission	Challenging behaviour	Resident carer	% Appropriateness score
1	Depression/anxiety	Risk of deliberate self-harm	Low	Yes	73.8
2	Depression/anxiety	Risk of deliberate self-harm	Low	No	95.4
3	Depression/anxiety	Risk of deliberate self-harm	Medium	Yes	56.0
4	Depression/anxiety	Risk of deliberate self-harm	Medium	No	78.9
6	Depression/anxiety	Other risks	Medium	Yes	26.2
7	Depression/anxiety	Other risks	Medium	No	54.4
9	Depression/anxiety	Behaviour management related	Medium	Yes	48.0
10	Depression/anxiety	Behaviour management related	Medium	No	28.3
16	Depression/anxiety	Assessment/treatment/check medication	Medium	No	33.3
21	Organic	Other risks	Medium	Yes	35.7
22	Organic	Other risks	Medium	No	41.3
26	Organic	Behaviour management related	Medium	Yes	23.8
28a^a^	Organic	Behaviour management related	High	Yes	28.6
28b^a^	Organic	Behaviour management related	High	Yes	53.8
29	Organic	Assessment/treatment/check medication	Medium	Yes	50.0
31	Organic	Assessment/treatment/check medication	High	Yes	11.8
40	Other	Behaviour management related	Medium	Yes	44.4

^a^People in case types 28a and 28b were admitted from home and care homes respectively

Thirty-eight staff participated in the care-planning workshops, and each vignette was considered by at least 17 individuals. Nurses were the most frequently represented discipline, but other participants included consultant psychiatrists, other doctors, occupational therapists and service managers. Equal numbers of staff worked in community and inpatient settings.

Of the 17 vignettes, that representing case type 2 (people with depression or anxiety whose admission was precipitated by the risk of self-harm) was considered most appropriate for inpatient admission, with an appropriateness score of 95.4 %. Indeed, 20 of the 22 practitioners who rated this vignette thought the person depicted was completely appropriate for inpatient care. In contrast, no-one considered case types 26 or 31 to be definitely suitable for admission, whilst the appropriateness scores for the five case types considered least appropriate for hospital care (the marginal inpatient case types) ranged from 11.8 to 28.6 %. Together these captured just over 18 % of admissions. Three predominantly or exclusively represented people admitted from home (6, 10 and 28a), and two admissions from care homes (26 and 31). The need for behavioural management was the main reason for admission in three of the five types (10, 26 and 28a).

### Proposed care plans

Ten different care packages were proposed for the marginal case types, ranging from one for case type 31 to three for case type 6, dependent on both the number of participating groups in each locality and the case types they deemed marginal. Each of the three plans for the two case types depicting care home residents involved input from a specialist care home support team, whilst those for people admitted from home drew on an intensive mix of primary care and mental health expertise, including frequent mental health support worker input to assist people with their diet, medication, personal and social care needs. The need for a carer’s assessment with a view to the provision of ongoing support and assistance was also highlighted.

### Cost comparisons

Table 6 compares the estimated weekly costs of the community care arrangements proposed by the experts with those of the care patients actually received prior to inpatient admission. In order to compare like with like, only those individuals who lived in the care setting described in the vignette are included in this and the following analyses (i.e., the care home residents represented by case types 26 and 31 and the people at home represented by case types 6, 10 and 28a). Gaps in the data on actual service receipt mean the numbers in this table are small. However, two things are striking: the very high degree of variation in actual service costs within case types; and the extent to which the estimated costs of the recommended care packages exceed the median costs of actual service receipt. Indeed, in all bar one case the former are at least double the latter.

**Table 6:** Marginal inpatient case types: Estimated weekly costs of community care^a^ and extent of informal care

Case type (setting)^b^	Number in setting	Number for whom have information on services	Median cost of actual care	Mean cost of actual care	Standard deviation	Min – max cost of actual care	Cost of recommended care	Number receiving any informal care	Number receiving 21+ hours informal care per week
6 (H)	8	5	192	216	236	0 - 600	561	6	2
10 (H)	4	4	137	247	328	0 - 714	260	1	0
26 (CH)	10	5	57	228	280	11 - 619	211	N/A	N/A
28 (H)	14	7	36	163	328	0 - 900	269	11	7
31 (CH)	3	2	70	70	100	0 - 141	517	N/A	N/A

^a^£s per patient

^b^H = home; CH = care home

In Table 7, the weekly costs of the recommended community care packages are then compared with those of inpatient care, highlighting the very high costs of hospital support. Thus, although the suggested packages were considerably more expensive than the community care people actually received, they were substantially lower than the costs of hospital admission. Furthermore, despite the fact that the recommended care packages often involved multiple staff and intensive input, the vast majority of this difference was attributable to the relatively low costs of providing specialist mental health care in the community as opposed to in hospital. Social care costs were relatively low in both scenarios.

**Table 7:** Marginal inpatient case types: Estimated weekly costs of inpatient and recommended community care^a^ by cost sector

Case type (setting)^b^	Inpatient care		Recommended community care		Total cost difference^c^
	NHS mental health costs	SSD costs	NHS mental health costs	SSD costs	
6 (H)	2,193	73	442	119	−1704
10 (H)	2,193	73	260	0	−2005
26 (CH)	2,193	73	186	24	−2055
28 (H)	2,193	73	155	115	−1996
31 (CH)	2,193	145	508	9	−1821

^a^£s per patient. Adjusted weekly costs i.e., one-off components have been distributed over the median length of inpatient admission for each case type

^b^H = home; CH = care home

^c^Recommended option minus original option

**Fig. 1:**
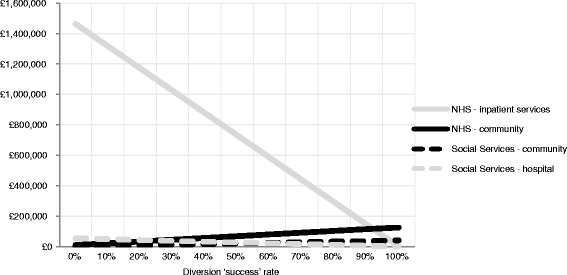
Marginal inpatient case types: The effect on different cost types of different diversion rates (£s per year)

Finally, Fig. 1 shows the potential aggregate savings that might be achieved if it proved possible to divert some or all of these admissions for a year. The key variables in these calculations are: i/ the number of patients represented by each case type over a 12-month period; ii/ the number of inappropriate admissions they would be expected to experience over a year; iii/ the average length of inpatient stay for each case type; and iv/ the number of days the alternative intensive community care packages would need to be employed. The first three of these variables are based on information from the service user profiling exercise, and the latter on information from the alternative care planning exercise. Thus if, for example, it proved possible to prevent all 20 of the admissions projected to be experienced by people in case type 26 over the course of a year, with each prevented admission releasing £18,813 from an average 62-day hospital stay, this could potentially amount to £376,260 per year. Indeed, assuming it was possible to divert all the patients in all five case types, local agencies might incur savings in the region of £1,300,000 (the difference between a 100 % diversion success rate and a 0 % diversion success rate), whilst even if it only proved possible to divert 50 % of each case type, savings of £680,000 could be attained. Diverting the care home residents represented by case types 26 and 31 could of itself potentially release £526,000.

## Discussion

The desire to curb rising health care costs has long-preoccupied many nations [16]. Against a background of increasing demand, the 2012 audit of investment in older people’s mental health services in England brings the scale of this challenge into focus, reporting a 3.1 % fall in real-term funding from the previous year [42]. This raises a number of important issues for local decision-makers, two of which have been addressed in the present work: the desire to prevent unnecessary hospital admissions and the determination of the optimal service mix.

Although previous studies have typically suggested that up to thirty per cent of adult mental health admissions may be inappropriate, this body of work is now several years old [51]. Moreover, little research has specifically explored the use of old age mental health beds. One point prevalence survey of older inpatients in the mid 1990s concluded that nearly a quarter no longer required acute hospital care [52]. However, community provision and admission thresholds are likely to have changed since then, whilst the authors did not consider how appropriate or necessary admission was in the first place. The fact that local practitioners in the current study had serious reservations about the appropriateness of more than a sixth of admissions is thus a significant finding, reinforcing the findings of the smaller BoC study mentioned above [30]. It is, however, important to note that these results do not automatically imply that current placement decisions are being made incorrectly, for these will have been constrained by the availability of alternative care. Rather, the findings identify the potential to divert a proportion of hospital admissions given an improved service mix, and provide commissioners with information on the needs and characteristics of those case types on whom any attempt to reduce the use of hospital beds might first focus. They also provide data on the mix of services necessary to effect such change.

With regard to the latter, the study suggests (perhaps surprisingly) that, at least for patients admitted from home, this would not necessarily require the development of new or innovative services, but rather more ‘standard care’, including intensive CMHT and primary care input. Indeed, most if not all of the proposed services were already available in the study catchment area, albeit they were often time-limited (support worker input), closed at weekends (CMHTs) or difficult to access. This desire to strengthen established services sits in contrast with growing calls for the development of a network of crisis resolution and home treatment services for older people [38, 39], emulating provision for working age adults [53]. Such services aim to provide an alternative to hospital admission by “intervening in the pathway between community-based referrers and inpatient care, providing robust assessment and gate-keeping of admissions” and typically operate a 24-hour service that seeks to resolve crises in people’s own homes ([54], p375). A recent report on adult mental health service development, however, noted that the creation of specialist teams with different access criteria can be confusing for referrers and lead to patients being passed from service to service [32]. Furthermore, there is as yet little robust evidence that crisis resolution and home treatment teams for older people with mental health problems can reduce hospital admissions, although feedback from early exemplars suggests a degree of perceived effectiveness [55].

Conversely, local staff perceived the development of specialist care home outreach as critical to the prevention of inappropriate admissions from care homes. Such services were seen to have the potential to undertake detailed behavioural and functional assessments that could act as the basis for person-centred interventions, and to serve as a single point of reference and support for care home staff. The external experts also stressed the relative advantage of such teams over the input of multiple professionals from different organisations, whilst whatever form such provision takes, an advantage of focusing on this client group is that the resources employed in preventing one admission should also prevent future admissions as care home staff gain skills and knowledge.

Whilst the results are encouraging in highlighting the potential for change, there are a number of reasons to treat the reported figures with caution. First, it should be noted that the given costs are all estimates (albeit sensitivity analysis suggested that even allowing for considerable uncertainty in the quantity and cost of resources, the general picture remained unchanged i.e., the cost of inpatient care was significantly higher that of the proposed community alternatives). Second, in considering only public expenditure, the study will have significantly underestimated the real costs to society of supporting people at home, including the costs of the substantial assistance provided by informal carers. Third, although the analysis identified a number of potentially inappropriate admissions to hospital, no information was collected about the extent of unmet demand. It is thus possible that other people, currently supported in the community, might be more appropriately admitted to hospital. Fourth, as in other BoC analyses, no account was taken of the need for parallel funding whilst community services are developed, a key ingredient in changing service provision. Fifth, in order to release significant savings, the number of hospital admissions prevented would need to reach a critical mass, so as to facilitate the closure of beds or wards. Indeed, it is not clear that even if it proved possible to prevent all 39 marginal admissions in this study, this crucial threshold would be reached, particularly given that the study was conducted across two Trusts. That said, the same organisations’ mental health services for working age adults already share wards, and the decision to focus the analysis on the five least appropriate case types for admission was a relatively conservative one, with the existence of two additional case types with appropriateness scores of less than 40 indicating the potential for local decision-makers to be more ambitious if so desired.

Finally it should be noted that although the mix of services available in the study area and the demographic and clinical characteristics of the patient population appeared typical of provision nationwide [50], it is not clear to what extent this study’s findings can be generalised to other localities. There is thus a need for further BoC studies to be undertaken in a wider variety of areas. These should, ideally, utilise a full social costings approach, whilst the design would also be strengthened by the incorporation of robust evidence about the relative outcomes of marginal patients supported in different settings. In the light of this gap, the current study assumed that staff’s decisions about appropriate care were “predicated by assumptions about what outcomes are desired” ([56], p9). However, this does not diminish the need for controlled comparisons.

## Conclusions

This study details the needs and number of older people admitted to acute old age psychiatry beds in three areas of North-West England (key information for local commissioners) and highlights the potential for change. The results suggest that if enhanced community services were available, a significant minority of inpatients could be more appropriately supported in their own homes at a cost considerably lower than that currently incurred. Furthermore, such a shift would not precipitate any obvious trade-off between health and social care costs, but the substitution of intensive specialist mental health care in the community for current inpatient treatment.

Writing more than 15 years ago, Wistow [57] noted that the need to improve efficiency can provide a powerful incentive to reshape services where this appears to be a cost effective strategy. Nevertheless, in practice, the reconfiguration of services is more often informed by historical precedent and government policy than by evidence on costs *or* effectiveness [56, 58]. In this situation, the BoC approach, which allows local decision-makers to systematically explore the costs and consequences of different courses of action, facilitating dialogue between diverse providers and supporting informed decision-making reflecting local judgements [59], appears to have much utility.

## Additional file

Additional file 1:
**Example of a vignette.**

## Abbreviations

ADLs: Activities of daily living
BoC: Balance of care
CMHT: Community mental health team
NHS: National Health Service
