# Supplementary material for: Identifying alternatives to old age psychiatry inpatient admission: an application of the balance of care approach to health and social care planning
Source: BMC Health Serv Res. 2015 Jul 17;15:267. doi: 10.1186/s12913-015-0913-1 (PMC4504087; doi:10.1186/s12913-015-0913-1)
Supplement: Additional file 1: — Example of a vignette. [file 12913_2015_913_MOESM1_ESM.doc]

**Additional File 1. Example of a vignette**

**Sociodemographic information:** Mr A is a 76 year old gentleman who lives with his wife.

**Past mental health support:** Mr A was referred to the mental health service approximately 2 years ago after his wife expressed concern about his increasing forgetfulness and repetition. Further to assessment by a consultant psychiatrist he was diagnosed with a mixed dementia and was seen briefly by the CMHT who provided his wife with information about dementia and local sources of help. Four weeks ago he was re-referred following concerns about his increasingly challenging behaviour and his medication was reviewed. There were also plans to instigate a behaviour management programme, and his wife has been keeping a diary of his presentation.

**Presentation immediately prior to/on admission**

**Activities of daily living:** Mr A is very active and has no problems mobilising in or outdoors. He is, however, occasionally incontinent of urine. Whilst he can physically wash, dress and feed himself (and has a good appetite), he needs much encouragement to change his clothes and can rarely be persuaded to have a bath.

**Physical health:** Mr A has high blood pressure and has previously been treated for prostate cancer. However, his current health is described as fair.

**Cognition/Mood:** As above, Mr A has dementia, now moderate-severe in degree. Whilst he has obvious short-term memory problems and sometimes struggles to express himself, his family can generally understand what he says. There is no indication that he is clinically depressed.

**Behaviour:** Mr A generally appears restless and agitated and often shouts at the television, seeming to think that the presenters are talking about him. At such times he can become very angry, and it is not uncommon for him to throw things at the television set, whilst if his wife tries to distract him, he then becomes angry at her. Although he previously slept well, in recent months he appears to have lost all sense of night and day, and is regularly up and about in the night, moving things from place to place. He does not, however, try to leave the house.

**Support immediately prior to admission:** Mrs A is her husband’s main carer and undertakes all the household tasks as well as providing a high degree of support and reassurance for her husband. Her own physical health is frail, however, and she is clearly very stressed by his recent behaviour. They do not receive any formal social care.

**Reasons for admission:** Mr A’s behaviour is causing concern and there are fears that he may, in anger, accidently harm his wife or himself. It is also thought that an inpatient admission would offer the opportunity to review his medication and future care needs.
